# Comparative study of paediatric prescription drug utilization between the spanish and immigrant population

**DOI:** 10.1186/1472-6963-9-225

**Published:** 2009-12-08

**Authors:** Luís A Gimeno-Feliu, Javier Armesto-Gómez, Rosa Macipe-Costa, Rosa Magallón-Botaya

**Affiliations:** 1 Aragonese Primary Care Research Group - Research Network on Preventative Activities and Health Promotion (redIAPP), Centro de Salud Arrabal, Gracia Gazulla 16, 50015 Zaragoza, Spain; 2 Aragon Health Sciences Institute, Avda Gomez Laguna 25, planta 11, 50006 Zaragoza, Spain; 3 Aragonese Health Service, Paseo María Agustín 16, 5001 Zaragoza, Spain

## Abstract

**Background:**

The immigrant population has increased greatly in Spain in recent years to the point where immigrants made up 12% of the infant population in 2008. There is little information available on the profile of this group with regard to prescription drug utilization in universal public health care systems such as that operating in Spain. This work studies the overall and specific differences in prescription drug utilization between the immigrant and Spanish population.

**Methods:**

Use was made of the Aragonese Health Service databases for 2006. The studied population comprises 159,908 children aged 0-14 years, 13.6% of whom are foreign nationals. Different utilization variables were calculated for each group. Prescription-drug consumption is measured in Defined Daily Doses (DDD) and DDD/1000 persons/day/(DID).

**Results:**

A total of 833,223 prescriptions were studied. Utilization is lower for immigrant children than in Spanish children for both DID (66.27 v. 113.67) and average annual expense (€21.55 v. €41.14). Immigrant children consume fewer prescription drugs than Spanish children in all of the therapy groups, with the most prescribed (in DID) being: respiratory system, anti-infectives for systemic use, nervous system, sensory organs. Significant differences were observed in relation to the type of drugs and the geographical background of immigrants.

**Conclusion:**

Prescription drug utilization is much greater in Spanish children than in immigrant children, particularly with reference to bronchodilators (montelukast and terbutaline) and attention-disorder hyperactivity drugs such as methylphenidate. There are important differences regarding drug type and depending on immigrants' geographical backgrounds that suggest there are social, cultural and access factors underlying these disparities.

## Background

We have witnessed a profound transformation in Spanish society in recent years resulting from the large increase in the immigrant population, most of whom have come from low-income countries [1-4]. Contrary to the situation a number of decades back, Spain is now considered a destination country for migratory flow.

According to Spanish National Institute of Statistics data taken from the Spanish Population Register on 1 January 2008, immigrants make up 12.7% of the total population of Spain. Immigrants comprise 12.46% of the child (≤14 years of age) population in Spain. In 2008, immigrants in the region of Aragon, where the study was made, accounted for 13.14% of the total population and 13.38% of the paediatric population

Spain is the European Union country that received the largest number of immigrants in 2007. The number of resident foreign nationals has multiplied by 3 in the last 6 years. It is clear that an increase of this scale in the immigrant population in such a short period of time involves adapting and reorganizing a number of aspects of the host society. This occurrence requires the destination society to undertake preparations in many aspects, among which are the public health service and health care. Awareness of the habits and needs of this population group is therefore of great interest so that resources may be planned and the necessary measures taken in order to for them to be faced in the best possible conditions [1,4-9].

Health care for non-EU foreign nationals in Spain is regulated by law and is guaranteed for foreign nationals, regardless of their residency status, in the same conditions as Spaniards [10]. Therefore, all children with foreign national status are covered by public health care, which is the same as that of the Spanish population.

Health care professionals and the public at large are generally unaware of the health care needs of, and services provided to, the immigrant population [5,7,11]. However, immigrants are believed to contribute to greater utilization of health care resources resulting in overcrowded health centres and accident and emergency services, and they are believed to have higher utilization of prescription drugs [12-15].

Despite the increase in recent years in the number of studies directed at analysing the health of immigrants, these studies tend to focus on medical aspects such as the prevalence of different diseases and conditions among foreign nationals, care procedures to be applied and mortality rates, among others [2,3,16-20]. However, there are very few studies aiming to discover the repercussions of providing health care to immigrants inside the health service, with a view to directing health policies and improving care. These studies additionally do not tend to focus on the paediatric population [5-7,11,21-25].

Pharmaceutical expense is one of the fastest growing components of the Spanish National Health Service budget. Subsidies for prescriptions dispensed in community pharmacies account for approximately 25% of total health care spending [26]. The expenditure generated by the immigrant population is therefore an important aspect to study when analysing the repercussions the immigration phenomenon is having on the public health system, given that the available information is very scarce. Additionally, as pharmaceutical expense is the result of prescriptions made by public health service doctors, this information is a good indication of the health care delivered.

In 2006, the population of Aragon (an autonomous region in northeastern Spain), all with access to public health care, was 1,273,662, of which 116,677 (9.16%) were foreign nationals. In the same year, 24,400,843 prescriptions were dispensed and billed to the Aragonese Public Health Service (SALUD) for a total value of €377,262,671. Public health system subsidies accounted for €341,731,076 of this amount.

Our study aims to provide insight into one of the least known aspects of the impact produced by the immigrant population on the Spanish health system - prescription drug utilization - compared to the Spanish population. The objectives of our work were:

1. to study the overall and specific differences in prescription drug utilization between the immigrant and Spanish paediatric population residing in a Spanish region; and 2. to understand the profile of drugs prescribed to both population groups, with regard to the main therapeutic groups prescribed and to the active ingredients that generate the greatest expense.

## Methods

This is a descriptive observational study on prescription drug utilization comparing the two different populations residing in Aragon: Spaniards and immigrants. In order to give an operational definition of immigrant, we have used the legal category of foreign national. This article makes use of both terms indistinctly [27]. Study population was the whole paediatric population of Aragon, both Spaniards and foreign nationals, with access to health care provided by SALUD accredited by their corresponding personal health insurance card. The period covered by the study was 1 January to 31 December 2006. This research has been aproved by Ethic Committe of Clinic Research of Aragon.

Pharmaceutical expense was analysed from the SALUD Pharmacy Service pharmaceutical billing database statistics for 2006 covering all publicly subsidised prescription drugs dispensed by pharmacies. These do not include drugs prescribed by private practices or those dispensed without prescription. Nor do they include drugs utilized in hospitals or dispensed there to outpatients (very specific treatments for particular diseases and conditions). Drugs corresponding to immunization campaigns are also excluded from this study. The data collected are cross-sectional. Long-term drug utilization patterns were not studied.

In order for their bills to be settled by the Pharmacy Service, pharmacists must use the computerized data-collection system to record the details of the prescribed drug (national drug formulary code), those of the prescribing doctor (health area identification code and registration number) and those of the patient for whom the drug is prescribed (personal identification code), among others.

By matching the personal identification code with the information from the health insurance card, which provides information on users' age, sex, country of birth and status (Spanish or foreign national), data can be obtained on utilization by age and sex of the local Spanish and immigrant population.

By matching the drug code with the drug formulary database, Digitalis, information can be obtained regarding the active ingredient, Anatomical Therapeutic Chemical (ATC) classification therapeutic main group and subgroup, the content in DDDs in each package needed to calculate the DID and whether it is a generic drug [28].

Statistical Analysis: Prescription drug utilization is compared by age, sex and foreign national or Spanish in the health card database. We applied the central limit theorem for large samples. The DID variable has a normal distribution and we were therefore able to use Student's t-test to compare these values between the immigrant and Spanish populations. P-values < 0.05 were considered statistically significant.

For each patient: DID, prescriptions, packages, health system subsidy, retail price of prescription drugs, utilization rate (patients utilizing the drug/drugs in 2006 compared to the total population by age at year's end) and percentage of generics utilized compared to total DDD.

Analysis was performed on anatomical groups, therapeutic groups and active ingredients with the purpose of ascertaining whether the differences in utilization occur in a similar manner in all prescription drug groups or whether, on the contrary, there is a different utilization profile. All of the prescribed and dispensed drugs and healthcare products were grouped according to the ATC classification system by anatomical main group, therapeutic main group, pharmacological and chemical subgroups, and active ingredients.

Inferential data analysis, which attempts to compare mean utilization per age and sex group, was performed using Student's t-test for independent samples. In order to be able to use this parametric contrast, we defined the central limit theorem, because this allowed us to work with a normal distribution when analysing the mean of a variable and, consequently, with parametric inference. The DID variable has a normal distribution and we were therefore able to use Student's t-test to compare these values between the immigrant and Spanish populations. P-values < 0.05 were considered statistically significant.

## Results

A total of 833 223 prescriptions made to children between the ages of 0 and 14 years were analysed. The reference population was 159 908 children residing in Aragon in 2006 of whom 13.6 (19 231) were foreign nationals. 24% of these foreign national children were born in Spain. The distribution by geographical background is as follows: 40% from South and Central America; 32% from Non EU- Europe; 21% from Africa; 8% of other origin.

**Figure 1:**
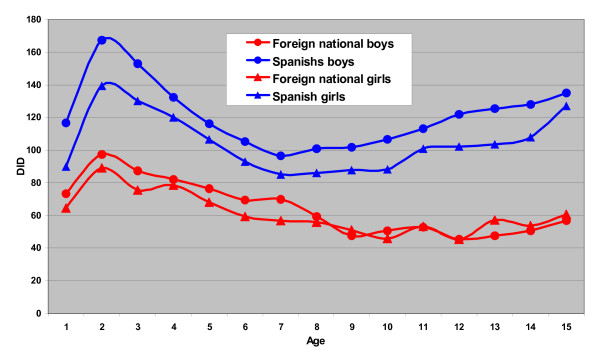
**Distribution by sex and age of the DID prescription**. Aragón, 2006

Figure 1 shows the differences in DID between children, with regard to age and sex.

Table 1 provides general information on prescription drug utilization for Spanish and foreign national children in Aragon regarding: DID, packages/person, expense/person, retail price/person, % co-payment - (retail price-expense/retail price) and utilization rate (percentage of the population that utilized at least one prescription in the year).

**Table 1:** Main indicators for prescription-drug utilization in spanish and foreign national children.

	Spaniards	Foreign Nationals	Difference Sp v. FN	p-value
**DID**	113.67	66.27	72%	<0.001
**Packages/person**	5.55	3.67	51%	<0.001
**Expense/person**	€ 29.38	€ 14.69	100%	<0.001
**Retail price/person**	€ 41.14	€ 21.55	91%	<0.001
**% co-payment**	29%	32%	-10%	<0.001
**Utilization rate**	74%	64%	16%	<0.001
**% DDD generics**	20%	18%	11%	<0.01

Rates adjusted by age and sex.

In all of the measurement units used - DDD, DID, packages, expense and retail price per person, prescription drug utilization by the Spanish population is far higher than that of the immigrant population (between 51% and 100%), with a great degree of significance.

Foreign nationals pay a higher co-payment percentage, no doubt owing to the lower value of their utilization of drugs for chronic conditions (with lower co-payment) than Spaniards. The prescription drug utilization rate for foreign nationals is only 16% lower than that of Spaniards, which means that the fundamental difference lies in the number of drugs consumed per person and the value of those drugs, rather than the number of children who utilized them.

Table 2 provides the percentage difference of Spaniards v. foreign nationals in the 18 anatomical and therapeutic groups studied, together with the general DID data previously presented.

**Table 2:** Difference in DID utilization between spaniards and foreign nationals by different anatomical and therapeutic groups.

ATC CLASSIFICATION CATEGORY NAME	CODE	Difference Sp v. FN	p-value
**TOTAL DID**		72%	<0.001
**RESPIRATORY SYSTEM**	R	93%	<0.001
**ANTI-INFECTIVES FOR SYSTEMIC USE**	J	65%	<0.001
**NERVOUS SYSTEM**	N	146%	<0.001
**SENSORY ORGANS**	S	65%	<0.001
**MUSCULO-SKELETAL SYSTEM**	M	45%	<0.001
**HORMONES FOR SYSTEMIC USE EXCL. SEX HORMONES**	H	104%	<0.001
**ANTIBACTERIALS FOR SYSTEMIC USE**	J01	72%	<0.001
**DRUGS FOR OBSTRUCTIVE AIRWAY DISEASES**	R03	142%	<0.001
**COUGH AND COLD PREPARATIONS**	R05	43%	<0.001
**ANTI-INFLAMMATORIES AND ANTIRHEUMATICS**	M01	44%	<0.001
**OPHTHALMOLOGICALS**	S01	61%	<0.001
**NASAL PREPARATIONS**	R01	146%	<0.001
**ANTIHISTAMINES FOR SYSTEMIC USE**	R06	113%	<0.001
**CORTICOSTEROIDS FOR SYSTEMIC USE**	H02	112%	<0.001
**PSYCHOANALEPTICS**	N06	668%	<0.001
**ANTICONVULSANTS**	N03	53%	<0.01
**ANALGAESICS**	N02	24%	<0.001
**PSYCHOLEPTICS**	N05	109%	<0.001

Rates adjusted by age and sex.

It can be observed that the differences in utilization are not homogeneous in the different drug groups. While the differences are relatively slight and below the mean for groups such as analgesics (N02) and anti-inflammatories (M01), there are very wide disparities in other groups such as bronchodilators (R03), decongestants (R01) and particularly psychoanaleptics (N06) and psycholeptics (N05).

Table 3 shows the analysis of active ingredients for which the active ingredients with the largest difference in utilization have been chosen, with a minimum utilization of 1 DID.

**Table 3:** Main active ingredients with highest DID and largest differences in utilization between spaniards and foreign nationals.

ATC	ACTIVE INGREDIENT	DID FN	DID SP	DIFF. DID	P-VALUE
N06BA04	**METHYLPHENIDATE**	0.50	3.93	687%	< 0.001
R03DC03	**MONTELUKAST**	0.47	2.28	385%	< 0.001
R03AC03	**TERBUTALINE**	0.59	1.98	238%	< 0.001
R01AD05	**BUDESONIDE (NASAL)**	1.45	3.79	162%	< 0.001
R01AD09	**MOMETASONE**	0.55	1.40	157%	< 0.001
J01DC02	**CEFUROXIME**	0.48	1.18	147%	< 0.001
H02AB07	**PREDNISONE**	0.61	1.51	147%	< 0.001
J01DC04	**CEFACLOR**	0.44	1.05	138%	< 0.001
R03BA02	**BUDESONIDE (INHALATION)**	0.98	2.33	138%	< 0.001
J01FA09	**CLARYTHROMICINE**	0.52	1.22	134%	< 0.001
R03BB01	**IPRATROPIUM BROMIDE**	0.57	1.23	117%	< 0.001
R05CB01	**ACETYLCYSTEINE**	1.91	4.14	117%	< 0.001

Rates adjusted by age and sex.

Drugs for problems of Attention-disorder hyperactivity such as methylphenidate show extremely significant differences in utilization. In the bronchodilator group, montelukast and terbutaline show differences in utilization that are higher than the group as a whole (385% and 238% compared to 142% for the R03 group). Within the antibiotics group, cefuroxime, cefaclor and clarythromicine show differences of 147%, 138% and 134%, respectively, compared to 72% for the J01 group.

Within the immigrant population, important differences are seen in utilization according to the geographical background and place of birth of the children, as shown in Table 4.

**Table 4:** Differences in DID prescription-drug utilization by place of birth of foreign national children. Aragón 2006.

GEOGRAPHICAL BACKGROUND	DID FN	DID SP	DIFF. DID	P-VALUE
**FOREIGN NATIONAL BORN IN SPAIN**	85.92	113.67	32%	<0.001
**SUB-SAHARAN AFRICA**	71.97	113.67	58%	<0.001
**NORTH AFRICA**	64.52	113.67	76%	<0.001
**EASTERN EUROPE**	56.50	113.67	101%	<0.001
**LATIN AMERICA**	55.13	113.67	106%	<0.001
**ASIA**	49.70	113.67	129%	<0.001
**WESTERN EUROPE AND NORTH AMERICA**	48.08	113.67	136%	<0.001

Rates adjusted by age and sex.

Certain heterogeneity was detected among immigrants themselves; children born in their countries of origin utilize fewer prescription drugs than those born in Spain. Children from western countries, Asia and Latin America have lower utilization rates than sub-Saharan and North Africans, although it will be necessary to study their qualitative utilization in greater depth in order to understand this phenomenon.

## Discussion

The results presented in this study are quite clear and consistent: prescription drug utilization is much greater in Spanish children than in immigrant children. In a National Health Service with universal child cover, despite what is thought, conveyed [29], and often appears in different media, our study shows that the utilization of prescription drugs by the immigrant paediatric population is far lower than that of the Spanish population. This has been demonstrated in the measurement of DID, total expense and number of packages consumed. Other studies had demonstrated this low utilization of services, although not all of them had given in depth study to drug utilization [5,7,22,30-33]. It has also been shown that this difference occurred in all therapeutic groups and main active ingredients although with huge variability.

Because the characteristics of Aragon are similar to Spain in distribution of resources, health system management, immigration population frequency, and social and demographic characteristics, our data may be generalizable for the entire country.

If we assume that the immigrant population has a socio-economic level that is lower than the Spanish population, it would seem that immigrants should have lower levels of health [34-36]. The low drug utilization found in our study could respond to the classic "Inverse Care Law" described by Hart in 1971 (patients receive medical care in inverse proportion to their need) or to their level of health being higher than expected, or to a combination of both [37]. There are a number of hypotheses explaining such striking differences.

One example is **accessibility**. There may be lower access for immigrant children, meaning they go to the doctor less regularly. However, there is no legal reason for this because the law guarantees their right to be attended in equal conditions. The Rivera Study concluded that there were no significant problems of access in Spain [7], but other authors have concluded that there are different barriers of culture, language, legal status, lack of familiarity with the health care system, and others that are related to work and timetables [38].

In order to explain this further, it would be of great use to have information on the utilization of the different health services by these patients. Data on frequency of consultations are disputed and new studies would be necessary to prove this point, but it would seem that immigrants make fewer consultations than Spaniards [5,7,11,32,33,39,40].

This factor would justify the lower overall prescription drug utilization, but not the important differences existing in the utilization profile.

This study only evaluates drugs prescribed by the public health services and does not include those dispensed with **private medical prescriptions **or those acquired over the counter without prescription, a situation that is quite common in paediatrics. It is estimated that of the total Spanish pharmaceutical market handled through the Pharmacy Service in 2006, 75.4% corresponded to the Spaninh National Health Service [41]. If this private utilization were taken into account, it would widen even further the differences between the two population groups, given that it is likely that immigrants would make less use of these services.

The cost of **co-payment **in Spain is very low and we do not consider this to be a reason that justifies the differences. In the analysis of utilization shown in Table 3, it could be suspected that the part to be paid by patients for some active ingredients influences patients with lower purchasing power to not accept having to pay a higher proportion of the price of a drug, therefore dissuading the doctor to prescribe it.

However, methylphenidate, the most utilized brand, has an extremely low price to be paid by patients, and both montelukast and terbutaline have a reduced co-payment scale - 10% of the total - with a maximum of €2.64 per package. In the case of the antibiotics cefuroxime, cefaclor and clarythromicine, the part to be paid by the patient is higher than for most drugs routinely prescribed in paediatrics such as amoxicillin and amoxicillin with clavulanic acid, with a lower cost per treatment, although these are acute treatments with a total cost that can never be high. Other factors may also be present in their being prescribed.

There are numerous studies pointing out that **immigrants usually have a better level of health **than their host populations because the healthiest are more likely to migrate ("healthy migration effect") and this population group has better lifestyles. These studies have been made on general populations and it is doubtful whether they can be applied to the paediatric population that has not undergone the selection process involved in emigrating to another country [5,7,39,42-47].

Some **cultural and sociological issues **arise when we analyse the enormous differences in the utilization of bronchodilators and Attention-disorder hyperactivity drugs (Table 3): Should we be considering that there is lower incidence of Asthma or Hyperactivity disorders in the immigrant population? Are we confirming the hygienist theories regarding the origin of asthma? Do immigrants with these conditions not receive treatment? Are Spanish children being overdiagnosed or overtreated for these conditions? Are these drugs being put to the correct use for which they were approved? Why do Spanish children utilize the latest Asthma treatments?

Prescription drug utilization does not only obey factors of health or illness. There are important sociological factors that influence it. The concept of and attitudes toward health and illness may be different in both groups. Our society is medicalized and seeks the solution to many of its problems in drugs. Different studies have confirmed that the self perception of good health is higher among immigrants than among locals [5,7,42].

Health care in most of the countries of origin of immigrants have shortcomings that often means medical treatment is given those processes considered most pressing, while people geneally turn to home remedies for different ailments. Over time, immigrants adapt and are assimilated into the culture of greater health-resource utilization of the host country, which would justify the differences found between children of immigrants born in Spain and those born in their countries of origin.

Likewise, we should also look at the concept of health and illness held by the immigrant population and the culture of health-resource utilization in their countries of origin that might justify a differential pattern of "felt morbidity". Health and illness are socially-constructed concepts and they vary in relation to the social and cultural context in which they are produced.

Taking into account a more biological concept, perhaps another motive could be health and the scale of needs. Consultations with immigrants tend to relate to more acute events; follow up is much more complex once the event for which the consultation was made has been overcome. Immigrants are very often unaccustomed to their children making regular visits to the doctor if they feel well. Adherence to chronic treatments is difficult. Preventive activities do not form a part of their requirements because they are not seen to be necessary, at least during their first years in Spain [48-50].

The finding that the differences are lower between drugs treating "acute" conditions (analgesics, anti-inflammatories and antibiotics) than drugs treating chronic conditions (Asthma and Hyperactivity) support this hypothesis.

## Conclusion

In conclusion, prescription drug utilization is much greater in Spanish children than in immigrant children, particularly with reference to bronchodilators (montelukast and terbutaline) and Attention-disorder hyperactivity drugs such as methylphenidate.

Prescription drug utilization by the immigrant paediatric population is much lower than for the Spanish population. Probably, cultural, sociological and accesibility factors are the factors that contribute more to these differences. Other studies will be needed to further analyse the disparities shown by drug subgroups, the idealness and suitability of prescription, the background of the immigrant population and frequency of consultations, among others, in order to assist us in ascertaining the causes of this situation.

## "WHAT THIS PAPER ADDS"

### What is already known about this topic?

There is not enough information about consumption of health resources from the immigrant paediatric population. Population studies could highlight disparities and explain variability in clinical practice.

### What this study adds?

Pharmaceutical consumption from immigrant children in a public health system such as Spain's, with universal health care, is very much lower than for native-born children. There are many differences, depending on country of origin and drug type. This fact entails relevant implications for the health planning and opens new research lines.

The **Corresponding Author **has the right to grant on behalf of all authors and does grant on behalf of all authors, an exclusive licence (or non-exclusive for government employees) on a worldwide basis to the BMJ Publishing Group Ltd and its Licensees to permit this article (if accepted) to be published in Journal of Epidemiology and Community Health and any other BMJPGL products to exploit all subsidiary rights, as set out in our licence http://jech.bmj.com/ifora/licence.pdf.

## Abbreviations

DDD: Defined Daily Doses; DID: DDD/1000 persons/day; SALUD: Aragonese Public Health Service; ATC: Anatomical Therapeutic Chemical.

## Competing interests

The authors declare that they have no competing interests.

## Authors' contributions

LAGF: conceived of the study, and participated in its design and coordination section. JAG: participated in the design of the study and performed the statistical analysis. RMC: participed in data analysis. RMB: participed in the design, and prepared the manuscript. All authors read and approved the final manuscript.

## Pre-publication history

The pre-publication history for this paper can be accessed here:

http://www.biomedcentral.com/1472-6963/9/225/prepub
